# Comparison between Procalcitonin, Brain Natriuretic Peptide, and Uric Acid in Children with Cardiomyopathy and Controls

**DOI:** 10.1155/2015/510450

**Published:** 2015-10-01

**Authors:** Noor Mohammad Noori, Maziar Mahjoubifard, Iraj Shahramian, Alireza Teimouri, Alireza Jahangirifard

**Affiliations:** ^1^Children and Adolescent Health Research Center, Zahedan University of Medical Sciences, Zahedan 9816743111, Iran; ^2^Department of Pediatrics, Faculty of Medicine, Zabol University of Medical Sciences, Zahedan 9816743111, Iran; ^3^Shahid Beheshti University of Medical Sciences, Tehran 1985717443, Iran

## Abstract

*Objective*. This study was performed to determine the level of procalcitonin, Brain Natriuretic Peptide (BNP), and uric acid in children with cardiomyopathy in comparison with controls and the association with echocardiographic findings. *Methods*. The levels of BNP, procalcitonin, and serum uric acid were measured and the amounts of biomarkers compared with echocardiographic findings. *Results*. In this study mean age of participants was the same (*p*=0.321). The majority of echocardiographic indices in left and right heart have different means in case and controls (*p*<0.05). Means of BNP, procalcitonin, and uric acid were 213.814 ± 309.601, 9.326 ± 3.881, and 6.846 ± 1.814 for case group and 2.76 ± 1.013, 1.851 ± 1.466, and 3.317 ± 0.924 for control (*p*<0.001), respectively. In the patients group there was relationship of Ross classification with BNP (*χ* ^2^ = 15.845, *p*<0.05) and with age (*χ* ^2^ = 8.946, *p*<0.05). For uric acid and procalcitonin no significant relationships were observed. *Conclusions*. procalcitonin, uric acid, and BNP had significant relationship with many echocardiographic findings in participants. For patients, procalcitonin did not show correlation. The severity of illness based on the Ross classification showed significant correlation with BNP level and age in patients.

## 1. Introduction

Cardiomyopathies, diseases of the heart muscles, primarily affect the left ventricle in which there is the main pumping chamber of the heart. Dilated cardiomyopathy (DCM) is recognized by left ventricular dilation that is concerned with systolic dysfunction. Diastolic dysfunction and impaired right ventricular function can occur. Left or right ventricular failure or both can be risk factors for affected individuals by DCMP. It may lead to ventricular arrhythmia, atrioventricular block, syncope, and sudden death [1]. The yearly incidence is 0.57 per 100 000 overall in children which is higher in males (0.66) than in females (0.47), in black (0.98) population than in white (0.46) per 100 000, and in babies younger than 1 year (4.40) than in children (0.34). In adults, the incidence is seven per 100 000 population [2, 3] and CMP is one of the main causes of heart failure in childhood [4]. NT-pro-BNP (Brain Natriuretic Peptide) is a prehormone synthesized in myocytes released cause of response to stress and increased in hypovolemia, renal failure status and by age [5]. BNP and NT-pro-BNP are extensively used for congestive heart failure diagnosis. These peptides may be useful for screening of asymptomatic high risk patients such as aged persons and patients with hypertensive, diabetic, and coronary artery disease [5]. BNP is a reliable and valuable test for diagnosis of functional and structural disorders of cardiovascular system but with cut-off point in children higher than that in adults [6]. The normal level of BNP is not able to confirm the existence of residual heart disease after cardiac surgery [7]. In cyanotic patients who have an increase in pulmonary artery pressure one observed an increase in serum uric acid level that is related to disease severity [8]. Hyperuricemia is an oxidative stress and is a good prognostic marker in patients with heart failure and is also a new independent risk factor for heart failure in young adults [9]. It was demonstrated that tubular secretion and uric acid clearance decrease in patients with decompensated severe heart failure and in patients with kidney dysfunction would result an increase in mortality rate [10]. Procalcitonin is a polypeptide made of 116 amino acids and is the precursor of calcitonin. The origin of procalcitonin in the inflammatory response is not yet completely understood. Procalcitonin is produced in the liver and peripheral mononuclear cells and modulated by cytokines [11]. The present study aimed to determine the level of procalcitonin, BNP, and uric acid in patients with dilated cardiomyopathy and in comparison with controls and assess their relationship with echocardiographic findings in participants.

## 2. Methods and Materials

This study was performed in two training hospitals located in Sistan and Baluchestan Province, Zahedan, Iran, from February 2014 to January 2015 among all patients with dilated cardiomyopathy. Patients' age ranged from 1 to 18 years. The study complies with the current ethical considerations. Informed consent was obtained from each patient's parents of the minors included in the study, and the study protocol conforms to the ethical guidelines of the 1975 Declaration of Helsinki as reflected in a priori approval by the institution's Human Research Committee. Exclusion criteria were hemoglobin less than 10 g/dL, endocrine, metabolic, and valvular disorders, dysrhythmia, and heart block. After considering exclusion criteria 37 patients were included in the study as case group and compared with 37 healthy children who matched in age and gender with case group. The control group was selected according to the normal status in clinical examination and echocardiography. Taking history, physical examination, CXRay, ECG, and echocardiography by MyLab 60 with transducer 3, 8 (made in Italy) were performed for all the participants. In this study, children weights for those over 2 years old were measured using RASA Mark made in Iran by an error of 100 g, while those for children under 2 years old were measured by MIKA Mark recumbent weighing scale made in Japan by an error rate of 10 g. In addition, under-2-year-old children's height was measured in the recumbent position by using a flat wooden calibration table, while that of the children above 2 years old was measured in the standing position with a scale ruler. Heart failure stratification was performed by modified Ross classification. The participant patients were normal in respect of Na, K, Ca, BUN, and creatinine. Three mL of blood was drawn from the patients while fasting at 8 am. Samples were centrifuged at 5°C with around 3000 g for 10 minutes. The separated serum was kept in −80°C refrigerator. The levels of BNP and procalcitonin were measured by ELISA kits. Serum uric acid level was measured by autoanalyser (NMCI). Echocardiography findings were measured by MyLab 60 made in Italy. Since the sample size is larger than 50, we use the Kolmogorov-Smirnov test for variables in the study. The null hypothesis for the test of normality stated that the actual distribution of the variable is equal to the expected distribution; that is, the variable is normally distributed. Since the probability associated with the test of normality for all variables except weight, left ventricular end-diastolic volume, fractional shortening, left peak E velocity, and right isovolumic contraction time is less than or equal to the level of significance (0.05), we reject the null hypothesis and conclude that these variables are normally distributed. Data analysis was performed by SPSS 20.0 software and, in the case of normality, independent *t*-test and one-way ANOVA were used and otherwise same nonparametric tests were applied. For the relationship between variables Pearson correlation was used. In all tests the value of 0.05 was considered for significant differences or correlations.

## 3. Results

In this study 74 children were recruited in equal case and control of 37 subjects. The participants' age ranged from 1 to 18 years with mean of 10.56 7 ± 5.5 and 12.135 ± 4.626 years for control and case, respectively, in which *t*-test results show nonsignificant difference (*p* = 0.321). The means of weight are 35.133 ± 18.471 and 28.7 03 ± 10.553 kg in control and case groups, respectively (*p* = 0.078). The means of height are 128.721 ± 29.231 and 127.405 ± 18.712 cm in control and case groups, respectively (*p* = 0.574).  Table 1 shows the results of independent *t*-test to compare means of echocardiographic findings of left and right heart in case and control groups. Means of left ventricular end-diastolic volume for case and control groups are 66.108 ± 22.129 and 39.279 ± 14.782 with significant different (*t* = −5.687, *p* < 0.001) and same trends for means of left peak E velocity with 85.974 ± 17.759 and 101.558 ± 19.648, respectively. The majority of echocardiographic indices in left (Table 2) and right (Table 3) heart have different means in case and controls in significant level. Mean rank of the left ventricular end-diastolic dimension of the case group is 42.568, while the participants in the control group have a left ventricular end-diastolic dimension score rank average of 23.433 with the significance <0.001 which shows statistically significant difference. Table 3 showed that the mean ranks of the right E/A velocity ratio were 33.216 and 34.967 in the case and control groups, respectively, with the *p* value of 0.714 in which there was no enough power to reject equality of mean ranks. Mean of Brain Natriuretic Peptide for case is 213.814 ± 309.601 versus control which is 2.76 ± 1.013 with the *p* value less than 0.001. Procalcitonin and uric acid have same patterns with levels of serum of 9.326 ± 3.881 versus 1.85 ± 11.466 and 6.84 ± 61.814 versus 3.317 ± 0.924 with the *p* values of 0.001 and 0.001 for case and control, respectively.  Table 4 shows Ross classification impact on age and the three biomarkers in the study. The results reveal that, amongst the biomarkers, BNP shows a difference in DCM severity levels. It shows that when DCM level increases the mean rank is increasing.  Table 5 shows significant correlation between age, uric acid, and BNP and some of echocardiographic parameters in patients' population. Patients' age has significant correlation with the most echocardiographic parameters except BNP (*r* = −0.05, *p* = 4.05), peak A velocity (*r* = −0.302, *p* = 0.069), ejection time (*r* = 0.291, *p* = 0.081), and interventricular septal dimension in diastole (*r* = −0.172, *p* = 0.309). Level of uric acid of patients has significant correlation with BNP (*r* = 0.707, *p* < 0.001) peak A (*r* = −0.364, *p* = 0.027) and IVSDD (*r* = 0.548, *p* = 0.000). BNP values have significant correlation with Et-p (*r* = 0.339, *p* = 0.050) and IVSDD (*r* = 0.493, *p* = 0.003).  Table 6 shows the correlation of echocardiographic parameters and biomarkers in participant's population. Participant age has significant correlation with LVDD (*r* = 2.28, *p* = 0.02), At-L (*r* = 0.37, *p* = 0.000), and Et-R (*r* = −0.25, *p* = 0.04). Weight of participants has significant mostly negative correlation with At-L (*r* = 0.31, *p* = 0.01), Et-R (*r* = −0.25, *p* = 0.04), Pep/et-R (*r* = −0.25, *p* = 0.04), LVPWD (*r* = −0.25, *p* = 0.04), and IVSS (*r* = −0.27, *p* = 0.03). Height like weight has negative or positive significant correlation with some of echocardiographic parameters or biomarkers, namely, EF (*r* = 0.24, *p* = 0.05), At-L (*r* = 0.27, *p* = 0.01), and dt-R (*r* = −0.25, *p* = 0.04). Procalcitonin has strong and significant correlation with the most echocardiographic parameters except At-R, dt-R, At-L, EF, and ivds. At the end of study, from 10 patients in group one, 2 patients died due to disease progression. All nine participants in the second group survived until the end of follow-up. In group 3 one patient died and one had heart transplantation (from 10 patients). And finally, for the fourth group, from eight patients, two received heart transplantation, one was with three-chamber pacemaker (died), and two individuals withdrew from the study.

## 4. Discussion

This study demonstrated that LVEDV (left ventricular end-diastolic volume), LVEDD (left ventricular end-diastolic dimension), LVESD (left ventricular end-systolic dimension), MPI (myocardial performance index), ICT (isovolumic contraction time), IRT (isovolumic relaxation time), E (peak E velocity), PEP/ET (preejection period/ejection time), ET (ejection time), IVSDD (interventricular septal dimension in diastole), LVPWDD (left ventricular posterior wall dimension in diastole), IVSDS (interventricular septal dimension in systole), and LVPWDS (left ventricular posterior wall dimension in systole) in left heart and MPI, IRT, DT (deceleration time), E, A (peak A velocity), PEP/ET, PEP, and ET in right heart were significantly different between two groups. We found that in patients with DCM the majority of echocardiographic parameters are increased in case group compared to control group. It also resulted that BNP and procalcitonin were increased significantly according to Ross classification but uric acid decreased nonsignificantly in the case group. Also some echocardiographic parameters were correlated with BNP and uric acid and also it was seen that BNP is a better marker compared with others due to association with severity and progression or regression of disease.

Increased BNP may approve the presence of DCM due to diastolic dysfunction and probably systolic dysfunction but without differentiation power between systolic and diastolic types. Accordingly, BNP has been approved by FDA for diagnosis of heart failure. Similarly we found association between BNP and MPI that shows systolic and diastolic dysfunction [12]. Jefferies and Towbin reported that DCM is characterized with left ventricular dilatation and is accompanied with systolic dysfunction. However diastolic dysfunction may also be seen and both ventricles may develop insufficiency and also there is mortality risk due to ventricular arrhythmia, atrioventricular block, syncope, and sudden death [1]. In our study for the case of patients, we found that BNP had strong relationship with three of echocardiographic findings and severity of illness in which consists with Jefferies' results. Law et al. reported NT-pro-BNP as a good biomarker for continuous left ventricle dysfunction in children with cardiomyopathy or myocarditis. However the normal level in improved children may not demonstrate any residual heart disease [6]. Their results are similar to our findings but we used BNP instead of NT-pro-BNP. Koch demonstrated that, among children with congenital heart disease, the plasma level of BNP is associated with ventricular function and reflects the ventricular overload injury. So the normal BNP may show compensatory phase [13]. Our results are similar to Koch's when we applied our study on children with DCM. In both studies the major problem is due to volume overload. Mariano-Goulart et al. reported that increased BNP level in patients with systolic ventricular dysfunction should be considered a risk factor for right ventricular dysfunction [14]. In our study it was also revealed that the BNP level was higher in the more severity of disease based on Ross classification. Kremastinos et al. reported that plasma levels of BNP and NT-pro-BNP are increased in major thalassemia patients and when left ventricular dysfunction is developed the predictive value of NT-pro-BNP is better than BNP for diagnosis of latent left ventricular diastolic dysfunction that is also increased by age [15, 16]. In our study a significant difference in mean age and BNP level was observed in various instances of severity of disease such that, in higher severity, the mean age and the mean BNP were higher. Noori and Rajaei reported that serum uric acid level in patients with DMC is increased and related to some echocardiographic findings. Another study by Cicoira et al. revealed that increased serum uric acid level is associated with diastolic dysfunction [17, 18]. In another study Noori et al. showed that in patients with beta thalassemia systolic and diastolic function of left and right heart were damaged. Also, it is recommended that in patients with major beta thalassemia without clear symptoms and signs of heart involvement the measurements of plasma level of BNP are necessary in addition to serial echocardiography in order to diagnose the early involvement of heart [19]. Krishnan demonstrated that hyperuricemia is an oxidative stress and is a good prognostic factor in patients with heart failure and also a new independent risk factor for heart failure in young patients [9] that agrees with our findings that showed increased serum uric acid level in case group. Niedner et al. found a relationship between BNP and severity of congenital heart disease. But in our study we found that increasing of BNP is correlated with severity of dilated cardiomyopathy [20]. Karoly Gombocz reported that procalcitonin plasma level had higher increase after operation in patients treated by gelatin compared to patients treated by dextran-70. The reason for this low increase in patients treated by dextran-70 would be considered to be due to systemic inflammatory response. An increase of procalcitonin in our patients compared to controls would be due to inflammatory process [11].

Sponholz et al. in a systematic review reported that, after uncomplicated cardiac surgery, procalcitonin levels increase to the peak level within 24 hours and return to normal levels within one week after operation. The degree of procalcitonin elevation depends on the intraoperative course and the type of the surgical procedure. Higher procalcitonin levels were observed in patients with a complicated postoperative course, with infection or sepsis syndromes compared to patients with an uncomplicated course. Procalcitonin is a useful test in differentiating acute graft rejection of heart and lung transplantation from bacterial and fungal, but not from viral, infections [21]. In our study the amount of procalcitonin in patient compared to controls increased which maybe was due to chronic inflammatory process. Madershahian et al. reported that procalcitonin increased with maximum concentrations on the second postoperative day. After the second day the levels decrease and will reach the normal concentration at the seventh day. Comparatively it is similar to our results that procalcitonin level increased but was nonsignificant when the degree of Ross classification is increasing. It revealed that when the severity of disease is enhanced the procalcitonin level rises [22].

## 5. Conclusion

The present research was performed on patients with DCM and showed that procalcitonin, uric acid, and Brain Natriuretic Peptide had significant relationship with the majority of echocardiographic parameters when we considered both case and control groups. Procalcitonin did not show any correlation with echocardiographic parameters in patients. The severity of illness based on the Ross classification showed significant and positive correlation with BNP level and age in patients.

## Figures and Tables

**Table 1:** Echocardiographic parameters of left and right heart.

Parameters	Group	Mean	SD	*t*-value	*p* value
Weight (kg)	Case	28.703	10.553	−1.7896	0.078
	Control	35.133	18.471		

Left ventricular end-diastolic volume (mL)	Case	66.108	22.129	5.687	0.000
	Control	39.279	14.782		

Fractional shortening (%)	Case	35.405	8.067	1.2972	0.199
	Control	33.333	3.736		

Left peak E velocity (cm/s)	Case	85.974	17.760	−3.4057	0.001
	Control	101.559	19.648		

Left peak A velocity (cm/s)	Case	55.377	15.504	−0.5643	0.574
	Control	57.545	15.814		

Right isovolumic contraction time (m/s)	Case	0.034	0.017	1.6933	0.095
	Control	0.0272	0.016		

**Table 2:** Results of the Mann-Whitney *U* test to compare the groups' values of left heart parameters.

Parameters	Group	Mean	SD	Mean rank	Sum of ranks	Mann-Whitney *U*	Sig. (2-tailed)
Height (cm)	Case	127.405	18.712	32.797	1213.5	510.5	0.574
	Control	128.7	29.2	35.483	1064.5		

Ventricular end-diastolic dimension (mm)	Case	46.584	7.713	42.568	1575	238	0
	Control	39.993	4.098	23.433	703		

Ventricular end-systolic dimension (mm)	Case	30.181	7.451	38.716	1432.5	380.5	0.028
	Control	26.517	3.23	28.183	845.5		

Ejection fraction (%)	Case	62.514	13.715	35.986	1331.5	481.5	0.353
	Control	63.533	5.507	31.55	946.5		

Myocardial performance index	Case	0.549	0.181	45.892	1698	115	0
	Control	0.325	0.051	19.333	580		

Isovolumic contraction time (m/s)	Case	0.03	0.01	43.865	1623	190	0
	Control	0.017	0.007	21.833	655		

Isovolumic relaxation time (m/s)	Case	0.107	0.026	43.23	1599.5	213.5	0
	Control	0.094	0.012	22.617	678.5		

Acceleration time (m/s)	Case	0.054	0.011	33.703	1247	544	0.888
	Control	0.054	0.008	34.367	1031		

Deceleration time (m/s)	Case	0.123	0.014	35.581	1316.5	496.5	0.456
	Control	0.121	0.015	32.05	961.5		

E/A velocity ratio	Case	1.657	0.757	28.757	1064	361	0.014
	Control	1.842	0.461	40.467	1214		

Left atrium/aorta ratio	Control	1.252	0.294	33.662	1245.5	542.5	0.875
	Case	1.203	0.15	34.417	1032.5		

Preejection period (m/s)	Control	0.128	0.156	44.703	1654	159	0
	Case	0.094	0.101	20.8	624		

Preejection period/ejection time (m/s)	Control	0.357	0.058	44.743	1655.5	157.5	0
	Case	0.294	0.039	20.75	622.5		

Ejection time (m/s)	Control	0.25	0.026	27.865	1031	328	0.004
	Control	0.263	0.02	41.567	1247		

Interventricular septal dimension in diastole (mm)	Case	6.319	1.294	40.243	1489	324	0.003
	Control	5.43	0.887	26.3	789		

Ventricular posterior wall dimension in diastole (mm)	Case	4.211	1.222	41.149	1522.5	290.5	0.001
	Control	3.343	0.507	25.183	755.5		

Interventricular septal dimension in systole (mm)	Case	9.276	1.761	39.365	1456.5	356.5	0.012
	Control	8.283	1.08	27.383	821.5		

Interventricular septal dimension in systole (mm)	Case	4.251	1.246	41.662	1541.5	271.5	0
	Control	3.363	0.524	24.55	736.5		

**Table 3:** Results of the Mann-Whitney *U* test to compare the groups' values of right heart parameters and biomarkers.

Parameters	Group	Mean	SD	Mean rank	Sum of ranks	Mann-Whitney *U*	*p*
Height (cm)	Case	127.405	18.712	32.797	1213.5	510.5	0.574
	Control	128.7	29.2	35.483	1064.5		

Myocardial performance index	Case	0.582	0.147	47.378	1753	60	0
	Control	0.316	0.05	17.5	525		

Isovolumic relaxation time (m/s)	Case	0.11	0.022	40.176	1486.5	326.5	0.004
	Control	0.096	0.012	26.383	791.5		

Acceleration time (m/s)	Case	0.134	0.197	30.068	1112.5	409.5	0.063
	Control	0.07	0.019	38.85	1165.5		

Deceleration time (m/s)	Case	0.128	0.012	38.689	1431.5	381.5	0.027
	Control	0.12	0.018	28.217	846.5		

Peak E velocity (cm/s)	Case	68.413	17.158	40.203	1487.5	325.5	0.004
	Control	57.706	14.139	26.35	790.5		

E/A velocity ratio	Case	1.408	0.478	33.216	1229	526	0.714
	Control	1.397	0.307	34.967	1049		

Peak A velocity (cm/s)	Case	50.366	13.852	40.203	1487.5	325.5	0.004
	Control	42.119	9.916	26.35	790.5		

Preejection period (m/s)	Case	0.085	0.015	43.054	1593	220	0
	Control	0.076	0.008	22.833	685		

Preejection period/ejection time	Case	0.34	0.06	45.973	1701	112	0
	Control	0.289	0.03	19.233	577		

Ejection time (m/s)	Case	0.246	0.021	27.365	1012.5	309.5	0.002
	Control	0.264	0.024	42.183	1265.5		

Procalcitonin (*μ*g/L)	Case	9.326	3.881	48.351	1789	24	0
	Control	1.851	1.466	16.3	489		

Uric acid (mg/dL)	Case	6.846	1.814	48.054	1778	35	0
	Control	3.317	0.924	16.667	500		

Brain Natriuretic Peptide (pg/mL)	Case	213.814	309.601	48.419	1791.5	21.5	0
	Control	2.76	1.013	16.217	486.5		

Age (year)	Case	12.135	4.626	36.122	1336.5	476.5	0.321
	Control	10.567	5.5	31.383	941.5		

**Table 4:** Ross classification impact on age and biomarkers in patients.

Parameters	DCM	Mean	SD	*N*	Mean rank	Chi-Square	*p*
Procalcitonin (*μ*g/L)	1	8.960	4.3254	10	15.2000	5.8195	0.1207
	2	8.022	3.9105	9	16.3333		
	3	8.925	2.4073	10	19.0500		
	4	11.750	4.3759	8	26.6875		

Uric acid (mg/dL)	1	6.930	1.9816	10	18.6000	0.1515	0.9850
	2	6.944	1.8602	9	20.1667		
	3	6.840	1.5932	10	18.9000		
	4	6.637	2.1427	8	18.3125		

Brain Natriuretic Peptide (pg/mL)	1	97.130	155.2863	10	12.7500	15.8453	0.0012
	2	76.656	76.4345	9	14.2778		
	3	133.050	102.7770	10	19.4500		
	4	614.925	452.6220	8	31.5625		

Age (year)	1	8.700	4.5473	10	11.0000	8.9456	0.0300
	2	12.556	4.5031	9	19.7222		
	3	14.400	4.1952	10	25.1000		
	4	13.125	3.4821	8	20.5625		

**Table 5:** Significant matrix correlation of echocardiographic parameters and biomarkers in patients' population.

Parameters	Age		Uric acid	
	PC	*p*	PC	*p*
Brain Natriuretic Peptide	−0.05	0.776	0.703	**0.000**
Left ventricular diastolic dimension	0.44	**0.007**	0.166	0.327
Ejection fraction	0.362	**0.028**	−0.03	0.86
Left isovolumic contraction time	0.327	**0.049**	−0.061	0.721
Left acceleration time	0.351	**0.033**	−0.053	0.758
Left peak A velocity	−0.302	0.069	−0.364	**0.027**
Right deceleration time	0.337	**0.041**	0.017	0.919
Ejection time	−0.482	**0.003**	−0.138	0.414
Left ejection time	0.291	0.081	0.072	0.674
Right ejection time	0.337	**0.041**	0.095	0.576
Interventricular septal dimension in diastole	−0.172	0.309	0.548	**0.000**
Ventricular posterior wall dimension in diastole	−0.334	**0.043**	0.222	0.186

**Table 6:** Matrix correlation of echocardiographic parameters and biomarkers in all population.

Echo parameters	Age		Procalcitonin		BNP		Uric acid	
	PC	*p*	PC	*p*	PC	*p*	PC	*p*
Left ventricular end-diastolic dimension	0.28	**0.02**	0.29	**0.02**	0.33	**0.01**	0.48	**0.00**
Left ventricular end-systolic dimension	0.17	0.16	0.18	0.15	0.21	0.09	0.34	**0.00**
Ejection fraction	0.22	0.07	0.00	0.98	−0.03	0.83	−0.08	0.54
Left myocardial performance index	0.14	0.27	0.39	**0.00**	0.15	0.25	0.49	**0.00**
Left isovolumic contraction time	0.15	0.22	0.44	**0.00**	0.47	**0.00**	0.43	**0.00**
Left isovolumic relaxation time	−0.18	0.16	0.27	**0.02**	0.10	0.44	0.21	0.09
Left acceleration time	0.37	**0.00**	−0.11	0.38	−0.12	0.33	0.01	0.92
Left peak E velocity	−0.17	0.18	−0.28	**0.02**	−0.21	0.09	−0.42	**0.00**
Right myocardial performance index	0.14	0.24	0.59	**0.00**	0.35	**0.01**	0.48	**0.00**
Right isovolumic relaxation time	−0.13	0.30	0.27	**0.03**	0.38	**0.00**	0.19	0.13
Right deceleration time	−0.07	0.56	0.11	0.36	0.20	0.12	0.26	**0.03**
Right ejection time	−0.25	**0.04**	0.27	**0.03**	0.03	0.79	0.19	0.12
Right acceleration time	−0.13	0.30	0.18	0.15	0.29	**0.02**	0.29	**0.02**
Left preejection period/ejection time	0.08	0.51	0.30	**0.01**	0.19	0.13	0.31	**0.01**
Right preejection period	−0.10	0.44	0.26	**0.03**	0.40	**0.00**	0.34	**0.00**
Right preejection period/ejection time	−0.10	0.42	0.32	**0.01**	0.36	**0.00**	0.39	**0.00**
Left interventricular septal dimension in diastole	−0.05	0.66	0.37	**0.00**	0.53	**0.00**	0.56	**0.00**
Left ventricular posterior wall dimension in diastole	−0.18	0.14	0.29	**0.02**	0.26	**0.04**	0.45	**0.00**
Left interventricular septal dimension in systole	−0.20	0.11	0.33	**0.01**	0.30	**0.02**	0.37	**0.00**
Left ventricular posterior wall dimension in systole	−0.15	0.21	0.35	**0.00**	0.32	**0.01**	0.47	**0.00**
